# Development of Functionalized Carbon Nano-Onions Reinforced Zein Protein Hydrogel Interfaces for Controlled Drug Release

**DOI:** 10.3390/pharmaceutics11120621

**Published:** 2019-11-20

**Authors:** Narsimha Mamidi, Aldo González-Ortiz, Irasema Lopez Romo, Enrique V. Barrera

**Affiliations:** 1 Tecnologico de Monterrey, Department of Chemistry and Nanotechnology, School of Engineering and Science, Monterrey 64849, Nuevo Leon, Mexico; aldogortiz@gmail.com; 2 Tecnologico de Monterrey, Department of Biotechnology, School of Engineering and Science, Monterrey 64849, Nuevo Leon, Mexico; iraromo18@gmail.com; 3 Department of Materials Science and NanoEngineering, Rice University, Houston, TX 77005, USA; barrera@rice.edu; 4 Department of Chemistry, Rice University, Houston, TX 77005, USA

**Keywords:** zein/poly 4-mercaptophenyl methacrylate-carbon nano-onions hydrogels, acoustic cavitation method, pH-responsive drug release, cytocompatibility

## Abstract

In the current study, poly 4-mercaptophenyl methacrylate-carbon nano-onions (PMPMA-CNOs = f-CNOs) reinforced natural protein (zein) composites (zein/f-CNOs) are fabricated using the acoustic cavitation technique. The influence of f-CNOs inclusion on the microstructural properties, morphology, mechanical, cytocompatibility, in-vitro degradation, and swelling behavior of the hydrogels are studied. The tensile results showed that zein/f-CNOs hydrogels fabricated by the acoustic cavitation system exhibited good tensile strength (90.18 MPa), compared with the hydrogels fabricated by the traditional method and only microwave radiation method. It reveals the magnitude of physisorption and degree of colloidal stability of f-CNOs within the zein matrix under acoustic cavitation conditions. The swelling behaviors of hydrogels were also tested and improved results were noticed. The cytotoxicity of hydrogels was tested with osteoblast cells. The results showed good cell viability and cell growth. To explore the efficacy of hydrogels as drug transporters, 5-fluorouracil (5-FU) release was measured under gastric and intestinal pH environment. The results showed pH-responsive sustained drug release over 15 days of study, and pH 7.4 showed a more rapid drug release than pH 2.0 and 4.5. Nonetheless, all the results suggest that zein/f-CNOs hydrogel could be a potential pH-responsive drug transporter for a colon-selective delivery system.

## 1. Introduction

Hydrogels are incipient three-dimensional (3D) networking structures that are able to swell in aqueous or non-aqueous fluids without dissolving. The hydrogels have been used in tissue engineering, drug release, material separation, and artificial organs, due to their excellent flexibility, high moisture content, and outstanding viscoelasticity [1]. Various hydrogels have been fabricated from synthetic and/or natural polymers with an emphasis on regenerative medicine, drug delivery, and tissue adhesives [2]. Such developed hydrogels are able to mimic the native extracellular matrix (ECM) and support cellular growth and tissue regeneration [3]. Besides, hydrogels are used in cell-matrix, 3D culturing cell-cell interactions, cellular proliferation, differentiation, and migration [4,5]. In this regard, naturally occurring biopolymers comprising hydrogels have potential advantages over synthetic polymers, including low cost, good cytocompatibility, less immunogenicity, degradability in physiological conditions, and wide availability [6]. Thus, several hydrogels developed from alginate, chitosan, hyaluronic acid, fibrinogen, collagen, and zein have been reported [7,8,9,10,11,12]. Consequently, it is vital to fabricate plant protein-derived hydrogels for biomedical applications. Zein is a natural plant protein and extracted from corn. Zein is hydrophobic and water-insoluble due to lack of prolamin [13,14,15,16]. Zein protein has been used in drug delivery, food packaging, and coatings [15,17]. However, there are some issues, such as shrinkage in water, low mechanical strength, and rapid degradation that impede the biomedical applications of pure zein. Therefore, several chemical modification and physical treatments have been developed to improve the mechanical strength, water-resistance, and plasticity of zein protein [18,19,20,21,22]. Moreover, zein was covalently functionalized with gold nanoparticles, polycaprolactone (PCL), and 3-glycidoxypropyltrimethoxysilane to improve mechanical and degradability, respectively [23,24,25]. In order to improve the above properties of zein, poly 4-mercaptophenyl methacrylate-carbon nano-onions were incorporated within zein protein to fabricate zein/f-CNOs composite hydrogels. 

Carbon-nano-onions (CNOs) are a class of carbon nanomaterials that contain concentric graphitic shells and are described by Ugarte in 1992 [26]. CNOs have been widely used in catalysis, supercapacitors, lithium-ion batteries, cell imaging, and diagnostic, therapeutic, and other biomedical applications because of their good physicochemical properties [27,28,29,30,31,32]. Among different carbon nanomaterials, CNOs are the most promising carbon material for biomedical applications because of their tolerance to transport in the circulatory systems with negligible toxicity and good cytocompatibility [31]. Moreover, several studies demonstrated that CNOs are highly biocompatible compared to MWCNTs, and CNOs showed less inflammation than CNTs [32]. Pristine CNOs, oxidized CNOs, and PEGylated CNOs are nontoxic and presented more than 85% of cell viability with fibroblasts [33]. Recently, ultra-high molecular weight polyethylene/4-mercaptophenyl methacrylate functionalized carbon nano-onions (UHMWPE/f-CNOs) nanocomposites were developed [34]. The mechanical, cytocompatibile, and thermal properties of the UHMWPE were significantly enhanced in the presence of 0.1 wt% of functionalized CNOs [34]. These outstanding results of f-CNOs motivated us to design and fabricate f-CNOs incorporated zein/f-CNOs hydrogels. Thus, it is of great interest to explore the application of CNOs as a second phase reinforcement in the fabrication of novel hydrogels.

The proper synthetic route of nanomaterials is essential in biomedical application, where the sonochemical method offers an easy path of synthesis. Sonochemistry is an emerging synthetic method to fabricate nanomaterials. Sonochemistry is a simple, facile, and short-time physicochemical method liable to the high-intensity ultrasound and acoustic cavitation phenomenon [35,36]. A sonochemical method has been used to develop several nanomaterials with an emphasis on controlled drug delivery [37,38]. Nonetheless, to the best of our knowledge, the fabrication of plant protein (zein)/f-CNOs composite hydrogels have not yet been established, until now, for controlled drug release. Thus, it is hypothesized that f-CNOs can uniformly disperse and reinforce within the zein matrix by sonochemical method. The homogeneous dispersion of CNOs not only improves the mechanical properties of zein/f-CNOs composite hydrogels but also enhances biodegradability, swelling, drug release, and cytocompatibility. 

The aim of the current study is to fabricate poly 4-mercaptophenyl methacrylated CNOs loaded zein protein hydrogels using acoustic cavitation technique. Besides, we will investigate the physicochemical properties, mechanical properties, drug release under physiological conditions, and cytocompatibility of hydrogels.

## 2. Experimental Section

### 2.1. Materials and Methods

All the reagents and organic solvents were purchased from commercial suppliers and used without further purification. Zein protein, methacryloyl chloride (MA), *N*-hydroxysuccinimide (NHS), 1-ethyl-3-(3-dimethylaminopropyl) carbodiimide (EDC), 5-Fluorouracil (5-FU), glutaraldehyde (GA), and 2,2′-Azobis(2-methylpropionitrile) (AIBN) were procured from Sigma Aldrich (St. Louis, MO, USA.). Osteoblast cells were obtained from the American Type Culture Collection (ATCC, Manassas, VA, USA.). Dulbecco’s modified Eagle’s medium/F12 without phenol red (DMEM/F12), phosphate buffered saline (PBS) pH 7.4, fetal bovine serum (FBS), penicillin/streptomycin, and trypsin were acquired from Gibco Invitrogen (Camarillo, CA, USA.). CellTiter96^®^AQueous One Solution Cell Proliferation Assay was bought from Promega, (Fitchburg, WI, USA.). LIVE/DEAD Cell Imaging Kit was purchased from Molecular Probes, Life Technologies Corp. (Carlsbad, CA, U.S.A.). 

### 2.2. Synthesis

#### 2.2.1. Preparation of Composite Hydrogels

The synthesis of poly 4-mercaptophenyl methacrylated-CNOs was accomplished according to the previous report [34]. 

##### Synthesis of CNOs-MP

Briefly, 100 mg of pristine carbon nano-onions were dispersed in 50 mL of anhydrous DMF for 30 min using an ultrasonic bath. Then, 350 mg of *N*-hydroxysuccinimide (NHS) and 350 mg 4-dimethylaminopyridine (DMAP) were added to the dispersion solution and further sonicated for 30 min. After that, 1-ethyl-3-(3-dimethylaminopropyl) carbodiimide (EDC, 570 mg) was added and sonicated for 30 min. Next, 100 mg of 4-mercaptophenol (MP) was added and stirring was continued for 48 h at 60 °C under N_2_. After the completion of the reaction (monitored thin layer chromatography), the stirring was stopped and the reaction mixture was cooled to ambient temperature. Next, the supernatant was discarded using centrifugation. The resulting black solid was thoroughly washed with DMF, methanol, DMF/triethylamine (9.9: 01), and ethyl acetate to obtain 4-mercaptophenylated CNOs (CNOs-MP).

##### Synthesis of CNOs-PMPMA

The mixture of 50 mg of CNOs-MP and 1.0 mL of diisopropylethylamine was dispersed in 50 mL of anhydrous tetrahydrofuran (THF) for 30 min using an ultrasonic bath. Then, 1.0 mL of methacryloyl chloride (MA) was added to the above mixture and stirred at room temperature for 24 h. Subsequently, the supernatant was discarded using centrifugation to attain a black solid. The resulted black solid was washed thoroughly with THF, dichloromethane, and HCl (0.01 M aqueous) to provide monomer CNOs-MPMA as a black solid. Then, 50 mg of CNOs-MPMA and azobisisobutyronitrile (AIBN, 1 wt%) were dissolved in 20 mL of anhydrous THF and sonicated for 30 min. Next, the reaction mixture was stirred at 70 °C for 48 h. Subsequently, the reaction mixture was cooled to ambient temperature and the supernatant was removed by centrifugation to attain CNOs-PMPMA as a black solid. The solid was washed with dichloromethane and diethyl ether and vacuum dried and stored in a desiccator until further use. The functionalized CNOs-PMPMA (f-CNOs) were characterized using ^1^H-NMR, Raman, TGA, and GPC analysis.

In order to prepare zein/f-CNOs hydrogels, initially, 2 mg/mL of f-CNOs was ultrasonicated for 30 min in water/1,4-dioxane (1:1) to obtain the homogenous dispersion. Then, 1.0 g of zein protein (in 10 mL of water/1,4-dioxane; 1:1) was added into the f-CNOs solution. The resulting reaction mixture was treated with GA (1%, *w*/*w*) as a crosslinking agent under three different reaction conditions as showed in the Table 1. 

To prepare the drug-loaded hydrogels, 10 mg/mL of 5-FU was added to the above solutions before the addition of crosslinker to incorporate the drug within the polymer matrix. This could prevent the surface drug loading, burst release, and aid the sustained release.

### 2.3. Characterizations of Composite Hydrogels

Scanning electron microscopy (SEM, ZEISS EVO^®^MA 25, Ostalbkreis, Baden-Württemberg, Germany) was used to study the surface morphological properties of composite hydrogels. For this, all the hydrogel samples were flash-frozen in liquid nitrogen and then fractured. Prior to the SEM analysis, the cross-sections of the freeze-dried hydrogel specimens were gold coated. Tensile strength and elongation at break testing were measured to evaluate the mechanical properties of composite hydrogels samples. A tensile testing machine (Instron 3365, Instron, and Norwood, MA, USA.) was used to measure the tensile properties of specimens. The size of the hydrogel sample was 33.0 mm × 6.0 mm × 2.0 mm, and the crosshead speed was 50 mm/min. 

To get the information about the functional groups presented in the hydrogel structure, the Fourier transform infrared (FTIR) spectroscopy (Perkin Elmer Universal ATR Sampling Accessory Frontier, Waltham, MA, USA) was used to scrutinize the zein/f-CNOs composite hydrogel specimens in the wavenumber range of 400–4000 cm^−1^ at room temperature. 

#### 2.3.1. Dynamic Light Scattering (DLS) and Zeta-Potential Experiments

DLS measurements were recorded using the Malvern Nano-ZS instrument and the data analyzed by Zetasizer software (version 7.12), Malvern Instruments Ltd., Worcestershire, WR14 1XZ, United Kingdom. 500 μg/mL of f-CNOs were probe sonicated in water and DMEM cell medium for 60 min. Then, the final concentration (500 μg/mL) of f-CNOs was diluted into 50, 25, 5, and 1 μg/mL in water and DMEM cell medium to measure the size of the particles. The dispensable zeta potential cuvettes were used to record the Zeta potential measurements. All the zeta measurements were run in triplicate per each sample and averaged to attain the final results. 

#### 2.3.2. Swelling of Hydrogels

Swelling ratio measurements of hydrogels were recorded by gravimetrically on a definite amount of dried hydrogel samples. Initially, the freeze-dried and pre-weighed hydrogel samples were immersed in DMEM (pH 7.4) at predetermined time intervals.

The swollen hydrogels were drawn from DMEM and the non-adsorbed medium was soaked mildly with filter paper and weighed with a microbalance. The swelling ratio (SR) was measured using the following equation:

(1) $$\mathrm{SR}=\frac{\left(Ws-Wd\right)}{Wd}\times 100$$

where *W_s_* is the weight of hydrogels at equilibrium state and *W_d_* is the weight of the hydrogels at the dry state. The swelling rate of the composite hydrogels was calculated according to the following equation

(2) $$v=\frac{\left(Wt2-Wt1\right)}{Wd\left(t2-t1\right)}$$

where *t*1 and *t*2 were the mean of the swelling time, and *W*_*t*1_ and *W*_*t*2_ were the weight of the sample at *t*1 and *t*2, and *W_d_* was the weight of dried hydrogels.

#### 2.3.3. In Vitro Degradation of Hydrogels

In vitro degradation of hydrogels was measured with respect to weight loss. For this, initially weighed hydrogel specimens (*W*_0_) were immersed in DMEM (pH 7.4) medium and incubated at 37 °C for 25 days. Then, the samples were taken out from the medium at predetermined time intervals, washed and dried in the desiccator for 12 h and weighed (*W_t_*). The weight loss ratio calculated as $100\times \frac{W0-Wt}{W0}$. The weight remaining ratio was calculated as $1-\left[100\times \frac{W0-Wt}{W0}\right]$.

#### 2.3.4. In Vitro Drug Release from Hydrogels 

UV-spectrophotometer (Agilent Technologies, 89090A) was used to measure the 5-FU release from the hydrogel specimens. For this, 30 mg of 5-FU-loaded hydrogel samples were immersed in 10 mL of DMEM (pH 2.0, 4.5, 7.4, and 9.0) and gently incubated at 37 °C. At predetermined time intervals, 2 mL of 5-FU released medium was collected and replaced with 2 mL of fresh DMEM medium to maintain the solution volume constant. The drug release was determined at λ_max_ = 265 nm to attain 5-FU concentration. The 5-FU release from hydrogel samples against time intervals was established. The pH-values (pH 2.0, 4.5, 7.4, and 9.0) of DMEM medium was adjusted with 1 M HCl or 1 M NaOH. Triplicate measurements were carried out. The drug release (%) was calculated from the following formula:

$$\mathrm{drug}\;\mathrm{release}\;\left(\%\right)=\frac{Mass\;of\;drug\;loaded\;Gel-Mass\;of\;drug\;released}{Mass\;of\;drug\;released\;}$$

#### 2.3.5. Cytotoxicity Evaluation of Composite Hydrogels

The human osteoblasts (bone-forming cells) were used to evaluate in vitro cytocompatibility of hydrogels. For this, osteoblast cells were cultured on the surface of the hydrogel samples. Cell viability and morphology were also studied. 

##### Cell Viability of Hydrogels

Cell viability was measured using CellTiter96^®^AQueous One Solution Cell Proliferation Assay. Initially, disk-shaped (~6.3 mm in diameter) hydrogels were prepared and then cut into thin sections and these hydrogel specimens were sterilized by ethanol (70% *v*/*v*) followed by UV irradiation. After that, the sterilized hydrogel specimens were decorated on a 96-well plate and then, human fetal osteoblastic cells (hFOB 1.19) at a density of 1 × 10^4^ cells per well (~3.12 × 10^4^ cells/cm^−2^) were seeded over the hydrogels. The non-adherent cells were removed on the next day. The cell number was calculated after 1, 2, and 3 days post-seeding, using CellTiter96^®^. Furthermore, the LIVE/DEAD Cell Staining Kit was used to measure the cell viability of hydrogels and the images were recorded using fluorescence microscopy. The tissue culture plate was used as a control in 96-well plates. The experiments were run in triplicate. 

##### Morphological Evaluations of Osteoblasts on Hydrogels 

Prior to cell seeding on 24-well plates, hydrogels were cut into a disk shape. After that, human fetal osteoblastic cells (hFOB 1.19) at a density of 1 × 10^4^ cells/mL were seeded on the surface of hydrogels in DMEM/F12 medium supplemented with 0.3 mg/mL of G418 disulfate salt, 2.5 mM of L-glutamine, 1% of penicillin/streptomycin and 10% of fetal bovine serum. The cells were incubated for 24 h in a humidified atmosphere at 5% CO_2_. Then, the medium was transferred from the well plates and washed several times with phosphate buffer solution. After 3 days of incubation, cell images were recorded by an optical microscope (Model IN200A-5M, Amscope, Chino, CA, USA).

### 2.4. Statistical Analysis

All the experiments were carried out in triplicate and quantitative data were presented as mean ± standard deviation (SD) with *n* = 3. Statistical analysis was determined by one-way analysis of variance (ANOVA) and Tukey’s post hoc tests using Minitab17 (Minitab, State College, PA, USA). *p* < 0.05 was considered statistically significant. 

## 3. Results

### 3.1. Synthesis 

**Figure 1:**
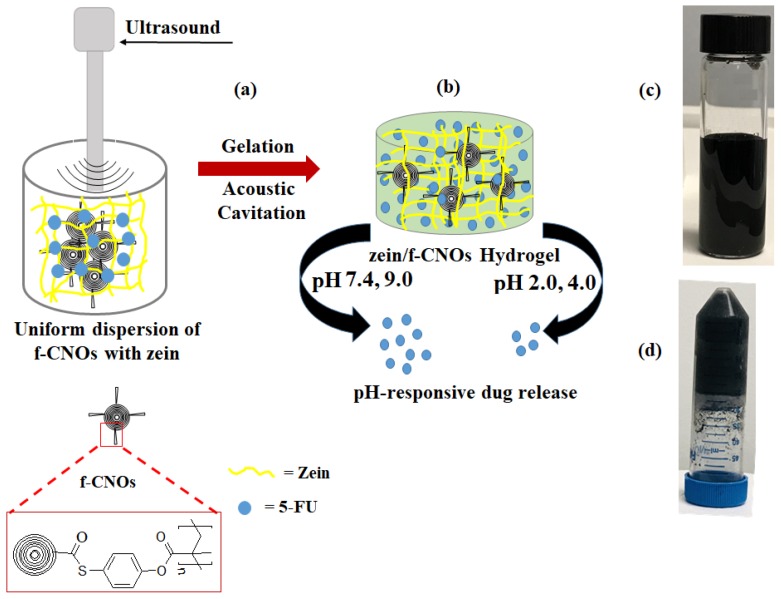
Synthetic illustration of (**a**) hydrogel preparation via acoustic cavitation, (**b**) cartoon diagram of hydrogel, (**c**) digital photograph of uniformly dispersed f-CNOs in DMEM after 1 year, and (**d**) digital photographs of fabricated UZCNOs hydrogel composite. f-CNOs indicates functionalized CNOs.

Recently, we synthesized poly 4-mercaptophenyl methacrylated CNOs (f-CNOs) starting from the thioester coupling of 4-mercaptophenol with COOH-CNOs followed by methacrylatation and polymerization [34]. Then, UHMWPE/f-CNOs nanocomposites were developed through reinforcing f-CNOs with long-chain UHMWPE. A small quantity of f-CNOs improved the mechanical and cytocompatibility of UHMWPE. The uniform dispersion of f-CNOs particles throughout the hydrogel matrix is necessary to achieve good physicochemical properties of zein/f-CNOs hydrogels. Therefore, f-CNOs were synthesized and zein/f-CNOs (UZCNOs) hydrogels were fabricated by reinforcing f-CNOs within the zein matrix through acoustic cavitation method (Figure 1a). The acoustic cavitation promoted 5-FU loaded UZCNOs hydrogels were crosslinked with GA. The UZCNOs hydrogels were pH sensitive, pH-responsive, and pH 7.4 and pH 9.0 exhibited quicker drug release than pH 2.0 and pH 4.5 medium (Figure 1b). Moreover, the synthesized f-CNOs were dispersed in physiological buffer (DMEM) or aqueous environment and stabilized uniform colloidal dispersion was observed over 12 months (Figure 1c). The digital photo of zein/f-CNOs hydrogel composite is presented in Figure 1d and the inverted image unveils the evidence of the gelation. 

### 3.2. Dynamic Light Scattering (DLS) and Zeta-Potential Measurements 

**Figure 2:**
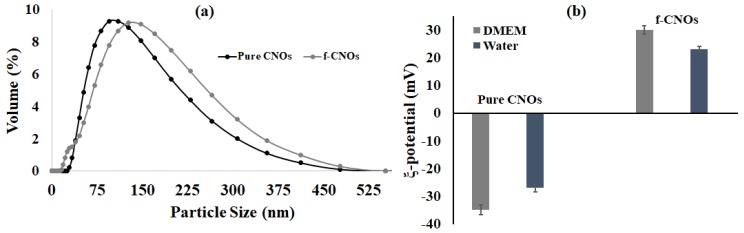
(**a**) Illustration of DLS particle size distribution, and (**b**) ξ-potential measurement of pristine CNOs and f-CNOs dispersions in DMEM.

The dispersion and stability of pure COOH-CNOs and f-CNOs were scrutinized under physiological environment by DLS measurements. The dispersion of pristine COOH-CNOs particles showed approximately 110 nm, whereas f-CNOs particles exhibited around 147 nm in DMEM (Figure 2a). The particle size of pristine CNOs and f-CNOs was not changed with the concentration and time, indicating a stabilized colloidal dispersion even at relatively high concentrations. Besides, zeta potential measurements of pristine CNOs and f-CNOs were measured in DMEM and water. The pristine CNOs exhibited ξ-potential values around −35 and −27 mV in DMEM and water, respectively, suggesting that they form uniform dispersions and that the COOH group has a substantial effect on the charge capacity of pristine CNOs (Figure 2b). Accordingly, positive (+ 30 and + 23 mV) ξ-potential values were observed in DMEM, and water, respectively (Figure 2b), indicating the chemical conjugation of the MPMA group on the surface of COOH-CNOs.

### 3.3. SEM Analysis 

**Figure 3:**
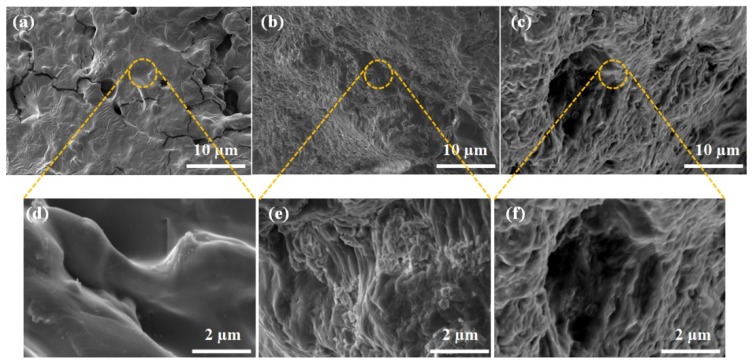
SEM image of composite hydrogel after freeze-drying (**a**,**d**) CZCNOs, (**b**,**e**) MZCNOs, and (**c**,**f**) UZCNOs, respectively. (**d**–**f**) Indicates the high magnification SEM images of CZCNOs, MZCNOs, and UZCNOs hydrogels, respectively.

The SEM analysis was performed to obtain microstructure morphologies of composite hydrogels and the resulting SEM images are illustrated in Figure 3. As shown in the SEM images, CZCNOs, MZCNOs, and UZCNOs hydrogel composites exhibited a porous and continuous structure. However, the porosity and internal morphology of hydrogels were reliant on the fabrication method. Particularly, the conventional thermal method provided CZCNOs showed constrained structure with low porosity (Figure 3a,d). On the other hand, MZCNOs hydrogel provided by microwave radiation showed a somewhat improved porosity (Figure 3b), and the porosity can be observed in the high magnification image of MZCNOs hydrogel (Figure 3e). Whereas UZCNOs hydrogel fabricated by acoustic cavitation exhibited sponge-like structure with good porosity, this porous morphology can restore the water uptake properties of the UZCNOs hydrogel (Figure 3c,f).

### 3.4. FTIR and Tensile Measurements

**Figure 4:**
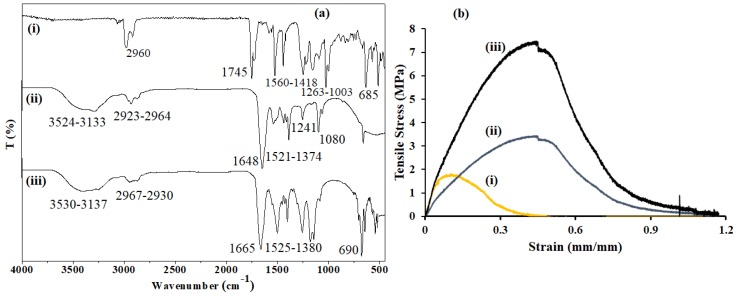
(**a**) FTIR spectra of (i) pure f-CNOs, (ii) pristine zein, and (iii) UZCNOs. (**b**) Tensile graph of (i) CZCNOs, (ii) MZCNOs, and (iii) UZCNOs hydrogels, respectively.

FTIR analysis was utilized to understand the possible interactions between zein and f-CNOs (Figure 4a). PMPMA chemically conjugated CNOs exhibited a major absorption band at 1745 cm^−1^ for C=O stretching and 1560–1418 cm^−1^ for vibrational starching of the phenyl ring. Besides, 1263–1003 cm^−1^ bands were absorbed for C–O–C stretching and 2960 cm^−1^ for aliphatic C–H stretching of PMPMA moiety. The f-CNOs also showed a sharp peak at 685 cm^−1^ for the C–H bending vibration (out of plane) of phenyl moiety. On the other hand, pristine zein showed a broad absorption band in the frequency range of 3524–3133 cm^−1^ for amide A (N–H stretching vibration) and 2964–2923 cm^−1^ for C-H stretching vibrations of aliphatic functional groups. In addition, the zein protein exhibited a band at 1648 cm^−1^ for amide-I and stretching vibration of C=O bond, and at 1551 cm^−1^ for amide-II and C–N bond, respectively [26]. Several absorption peaks of the N–H bending and C–N stretching of zein protein were detected at 1521–1374, 1241, and 1080 cm^−1^, respectively. As shown in Figure 4a, UZCNOs composite hydrogel showed a broad absorption band in the range of 3530–3137 cm^−1^, and 2967–2930 cm^−1^ for amide A (N–H stretching vibration), and C-H stretching vibrations of aliphatic functional groups, respectively. UZCNOs sample also showed the absorption peak at 1665 cm^−1^ for amide-I, and C=O stretching, respectively. The C–N bond and amide-II of UZCNOs hydrogels were detected in the frequency range of 1525–1380 cm^−1^. Furthermore, UZCNOs hydrogel showed the absorption bands at higher a frequency range for C–N stretching, and N–H bending, respectively. Notably, C–H bending (out of plane) of phenyl moiety (f-CNOs) was absorbed at 690 cm^−1^. 

The tolerance of high loads and sustained deformation of the hydrogel are important characteristics in drug delivery applications. Consequently, the mechanical properties of UZCNOs hydrogel composite were recorded and non-linear stress/strain curves are illustrated in Figure 4b. The mechanical properties of UZCNOs hydrogels were significantly (*p* < 0.05) enhanced with the addition of f-CNOs. Specifically, UZCNOs hydrogel composite showed 7.7 ± 0.25 MPa of tensile strength, whereas MZCNOs hydrogel composite exhibited 4.2 ± 1.12 MPa of tensile strength (Figure 4b). These results revealed that UZCNOs hydrogel composite displayed higher tensile strength and lower strain than MZCNOs hydrogel. This could be due to the crosslinking density, electrostatic interactions or hydrogen bonding between f-CNOs and zein protein under acoustic cavitation environment. In addition, the tensile strength of CZCNOs hydrogel was measured (0.98 ± 1.25 MPa) and compared with UZCNOs hydrogel. 

### 3.5. Swelling and Degradation Measurements

**Figure 5:**
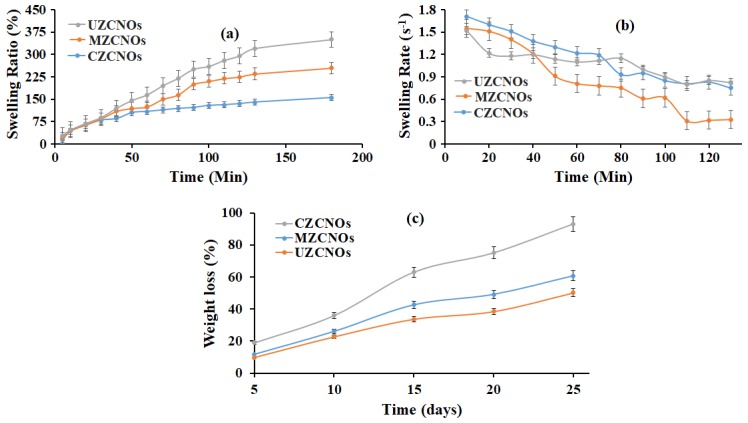
(**a**) Swelling ratio, (**b**) swelling rate, and (**c**) degradation curves of (i) CZCNO, MZCNOs, and UZCNOs hydrogels, respectively, in DMEM (pH 7.4) at room temperature.

Usually, the water absorption of the hydrogel can be influenced by the aggregate structure and chemical structure. Thus, the swelling ratio and swelling rate of the fabricated hydrogels with different methods were measured using the gravimetric method and the results were presented in Figure 5a,b, respectively. From Figure 5a, it can be seen that the percentage swelling of the composite hydrogels was more than 150%. Particularly, UZCNOs hydrogel synthesized by acoustic cavitation and MZCNOs synthesized by microwave radiation showed the higher mass swelling ratio (>255%), this indicates that f-CNOs have a detrimental effect on the swelling ratio. Besides, the swelling rate of hydrogels was measured and is illustrated in Figure 5b. According to Figure 5, the swelling rates of the all the composite hydrogels were gradually decreased and reached equilibrium values, which indicates that the swelling behavior of hydrogels could be controlled with a short period for zein/f-CNOs hydrogels. Subsequently, this can be a rapid absorbent.

The biodegradation characteristics of composite hydrogels display an important role in drug delivery and tissue engineering. Consequently, the degradation of hydrogels was measured in DMEM (pH 7.4) at 37 °C and the results are illustrated in Figure 5c. The CZCNOs hydrogel showed approximately 93% of degradation in 25 days of incubation. This could be due to the existence of weak electrostatic interactions and a difference in crosslinking density under the conventional method. On the other hand, MZCNOs and UZCNOs hydrogels showed sustained weight loss up to 25 days of incubation (Figure 5c). The UZCNOs hydrogel exhibited a lower degradation rate than MZCNOs hydrogels. Specifically, UZCNOs hydrogel showed around 50% of degradation in 25 days of study. This could be due to the strong electrostatic interactions and higher crosslinking density under acoustic cavitation. Whereas MZCNOs hydrogel exhibited approximately 61% of degradation in 25 days of study. 

### 3.6. PH-Responsive Drug Release

**Figure 6:**
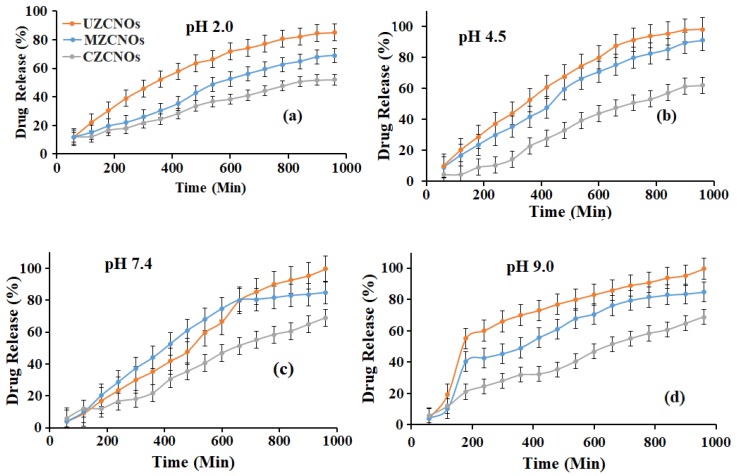
The graph illustrating the cumulative release of 5-FU from CZCNOs, MZCNOs, and UZCNOs composite hydrogels in DMEM at (**a**) pH 2.0, (**b**) pH 4.5, (**c**) pH 7.4, and (**d**) pH 9.0 at 37 °C, respectively.

In vitro release of 5-FU from composite hydrogels was performed by immersing the 5-FU loaded hydrogels in DMEM at pH 2.0, 4.5, 7.4, and 9.0. The drug release curves are presented in Figure 6. Initially, the 5-FU release from CZCNOs, MZCNOs, and UZCNOs hydrogels was measured at pH 2.0 over 15 days (Figure 6a). Under these conditions, composite hydrogels showed prolonged drug release. Particularly, the CZCNOs sample exhibited approximately 52% and the MZCNOs sample showed 69% of drug release over 15 days of study (Figure 6a). On the other hand, the UZCNOs sample showed 85% of drug release over 15 days at pH 2.0. 

To improve the drug release profile, pH 4.5 was used and measured the 5-FU release from composite hydrogel specimens (Figure 6b). As expected, CZCNOs hydrogel specimen exhibited around 62% of drug release over 15 days of study, which improved 8% of drug release at pH 4.5 (Figure 6b). The MZCNOs hydrogels sample showed around 91% of 5-FU release after 15 days of study and 20% of drug release was improved by changing pH 2.0 to pH 4.5., whereas the UZCNOs hydrogel sample synthesized by acoustic cavitation method exhibited approximately 94% of drug release at pH 4.5 over 15 days of incubation and reached plateau (Figure 6b). 

In addition, 5-FU release was carried out in DMEM at pH 7.4 at room temperature (Figure 6c). This pH environment is defensible to the normal cells and reduces drug loss during drug transportation. Besides, pH 7.4 can control drug release behavior of hydrogel specimens while they present at the cytoplasm of normal cells or intracellular environment. The CZCNOs hydrogel showed around 67% of sustained drug release over 15 days of study. The MZCNOs hydrogel exhibited 85% of sustained drug release, whereas UZCNOs hydrogel samples displayed approximately 97% of sustained drug release over 15 days of study and reached a plateau. 

Furthermore, 5-FU release was measured at pH 9.0 and the results are illustrated in Figure 6d. The UZCNOs hydrogel specimen showed approximately 52% of burst release on the third day of study. After that, around 99.9% of the drug was released in 15 days of study and reached a plateau (Figure 6d). On the other hand, MZCNOs hydrogels showed around 40% of burst release on the third day, followed by 85% of sustained drug release over 15 days of study, which is very similar to the pH 7.4 study (Figure 6d). Finally, CZCNOs hydrogels showed approximately 70% of sustained drug release over 15 days of incubation (Figure 6d). 

### 3.7. In Vitro Cytocompatibility Measurements 

**Figure 7:**
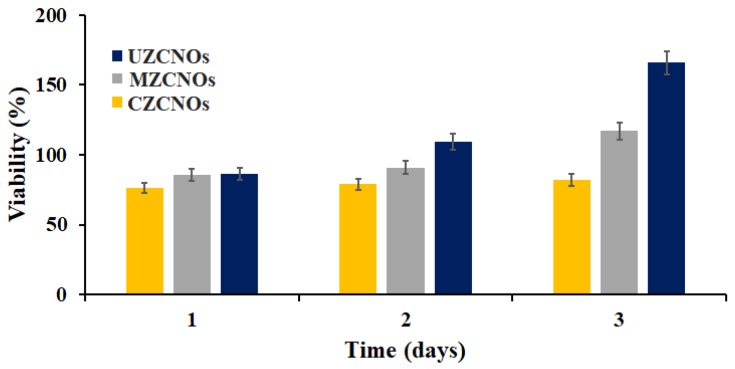
Cell viability of CZCNOs, MZCNOs, and UZCNOs composite hydrogels. Data represent mean ± SD (*n* = 3). Statistically significant difference (*p* < 0.05) was observed between the cell viability parameters of CZCNOs, MZCNOs, and UZCNOs samples.

The CellTiter96^®^AQueous One Solution was used to measure the cell viability of composite hydrogels and the results are depicted in Figure 7. The osteoblast cells were cultured on the pre-sterilized hydrogels specimens. On the first day of incubation, very similar cell viability was observed in CZCNOs, MZCNOs, and UZCNOs composite hydrogels. On the second day of study, MZCNOs, and UZCNOs hydrogel samples showed slightly improved cell viability, whereas CZCNOs hydrogel sample exhibited comparable viability on the same day of study (Figure 7). However, on the third day of incubation, the UZCNOs hydrogel specimen exhibited a better cell viability than the MZCNOs and CZCNOs hydrogel specimens. 

**Figure 8:**
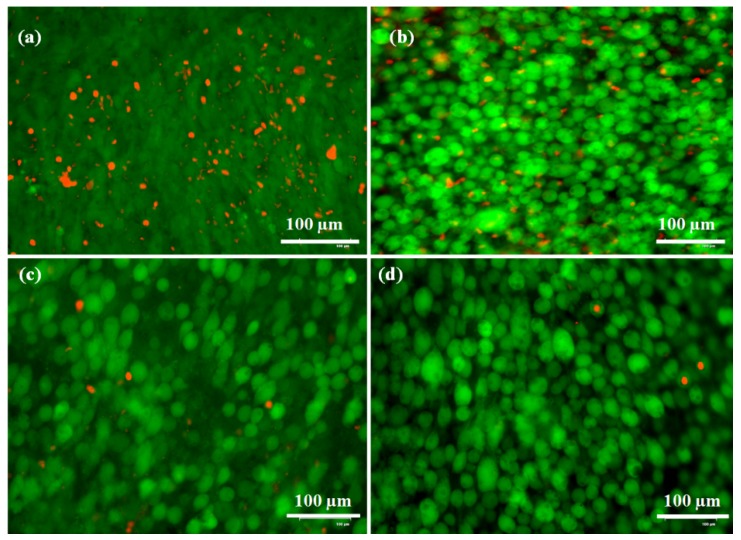
Optical images of osteoblast cells on the surface of zein/f-CNOs composite hydrogels after 3 days of study: (**a**) The control (tissue culture plate), (**b**) CZCNOs, (**c**) MZCNOs, and (**d**) UZCNOs; green indicates LIVE cells, and red indicates DEAD, respectively. Scale bar = 100 μm.

The LIVE/DEAD kit was also utilized to evaluate the cytotoxicity of f-CNOs in zein/f-CNOs composite hydrogels and the resulting optical images are depicted in Figure 8. The cell culture plate was used as a controller and it exhibited some DEAD cells after three days of study (Figure 8a). The CZCNOs hydrogel sample showed a decent percentage (77 ± 1.30%) of LIVE cells along with some of the DEAD cells (Figure 8b). The MZCNOs hydrogel sample displayed approximately 93 ± 0.81% of LIVE cells along with a small number of DEAD cells (Figure 8c). Interestingly, UZCNOs sample exhibited more than 97 ± 0.41% of LIVE cells (Figure 8d). 

## 4. Discussion

The homogeneous colloidal dispersion measurements suggested that the functionalized CNOs could stabilize in the aqueous environment. The acoustic cavitation wave or acoustic cavitation is the superlative stringent source of exfoliation of 2D nanomaterials. The fluctuation of pressure in the liquid environment generates the acoustic cavitation that creates bubble growth followed by bubble collapse and finally internal turbulence. This ultrasound energy renovates into high temperature and pressure. Ultrasound waves transfer through carbon nanoparticles (CNOs) which are held by weak interactions, including the Van der Waals forces and/or π–π stacking. Thus, acoustic cavitation is a good choice to achieve the uniform dispersion and stability of CNOs in the aqueous environment. Colloidal stable CNOs produced by acoustic cavitation are effective to enhance the electrical, tensile, and biocompatible assets of the nanocomposites. Consequently, uniformly dispersed and colloidal stabilized f-CNOs were reinforced with zein and fabricated zein/f-CNOs hydrogel composite through the acoustic cavitation method. Thus, the positive and negative ξ-potential values described the proton exchange phenomenon between the polar groups present on the surface of CNOs and the solvent system. When COOH-CNOs were dispersed in DMEM and water, CNOs groups were able to donate protons to the medium, exhibiting the negative ξ-potential values. On the other hand, chemically conjugated CNOs (f-CNOs) were unable to offer protons to the medium, switching the ξ-potential to positive values. The SEM analysis of UZCNOs hydrogel exhibited a sponge-like structure with porous morphology.

All these absorption bands of pristine zein were altered in the FTIR spectrum of UZCNOs hydrogel composite, suggesting that there were hydrophobic interactions or π-π stacking between the zein protein and f-CNOs. Thus, UZCNOs hydrogel composite showed a peak at 1665 cm^−1^ for C=O stretching vibration and amide-I, whereas, 1525 cm^−1^ for amide II and the C–N bond, respectively. Likewise, the N–H bending and C–N stretching peaks of UZCNOs hydrogel were shifted to a higher frequency range, which reveals that f-CNOs were completely blended within the hydrogel matrix. Thus, FTIR spectra show physicochemical interactions between zein protein and f-CNOs. The tensile results suggest that the UZCNOs hydrogel produced by acoustic cavitation has exhibited approximately seven times higher tensile strength than CZCNOs hydrogel produced by a conventional stirring method. Accordingly, the mechanical measurements reveal that the hydrogel fabrication method could be critical to obtaining enhanced tensile properties. 

The percentage of the swelling ratio of the CZCNOs hydrogel synthesized by the traditional stirring method was significantly lower than UZCNOs and MZCNOs (Figure 5a). This could be due to the difference in crosslinking density. By comparing the swelling results from Figure 5a, it can be revealed that the swelling ratio of CZCNOs hydrogel was remarkably lower than MZCNOs and UZCNOs hydrogels, which indicates that the crosslinking density was higher for hydrogels fabricated by radiation and acoustic cavitation. The UZCNOs hydrogels showed the lowest degradation in 25 days of incubation. This could be due to the existence of moderate electrostatic interactions and the difference in crosslinking density under the microwave method. Overall, the fabrication method had a considerable impact on the degradation measurements. 

The UZCNOs hydrogels exhibited higher drug release than MZCNOs and CZCNOs hydrogels at pH 2.0 and 4.5. This could be due to the π-π stacking and Van der Waal forces between hydrogels and 5-FU. These characteristics might have slowed the diffusion rate of 5-FU from the composite hydrogels. Besides, it is hypothesized that the solubility and diffusion of 5-FU dawdled in the presence of f-CNOs. Moreover, the fabrication method, lower swelling ratio, and mobility of f-CNOs played a key role in the drug release measurements. In addition, UZCNOs hydrogel showed improved drug release (97%) at pH 7.4. We relate this to the high swelling ratio of the hydrogels at pH 7.4. Furthermore, the 5-FU release rate is significantly proportional to the swelling behavior of the hydrogel network. Whereas at pH 9.0 we did not see a significant improvement in the drug release of MZCNOs and CZCNOs specimens by increasing from pH 7.4 to pH 9.0, however, the UZCNOs hydrogel sample exhibited burst release followed by sustained drug release up to 15 days of study. It could be due to the hydrophilic nature of 5-FU within the gel matrix, which led to faster diffusion from the hydrogel into the medium. 

The drug release results at pH 7.4 and 9.0 are attributed to π–π stacking of 5-FU molecules on the surface of composite hydrogels. 5-FU is a chemotherapeutic agent and is used clinically for colorectal carcinoma and there is a great need for colon-specific controlled delivery systems to treat directly at a disease site in the colon [39]. The results of in vitro 5-FU release from zein/f-CNOs suggest that the UZCNOs could be a prospective pH-sensitive transporter for colon-specific drug delivery. It is well known that the natural pH environment of the gastrointestinal tract differs from acidic (stomach) to slightly basic (intestinal). Particularly, the gastrointestinal tract increases its pH environment from the stomach (pH 1.4–3.0) to the terminal ileum (pH 7.5 ± 0.5). Such pH environment decreases to 6.4 ± 0.6 at the beginning of the colon, which slightly rises to pH 6.6 ± 0.8 in the middle of the colon and reaches 7.0 ± 0.7 in the left colon [40]. Therefore, it is important to consider the pH environment of the gastrointestinal tract while designing peroral dosage forms. The pH-responsive release of 5-FU from the UZCNOs hydrogels could indicate that zein/f-CNOs composite has an admirable protective effect for the oral delivery system of peptides and other drugs, which are easily ruined by gastric acid. Thus, the loaded drug can be released in a lesser amount from the hydrogel as it travels through the stomach. After reaching the colon, a significant amount of drug retained in the gel matrix could be released from the hydrogel. 

Besides, UZCNOs hydrogels displayed higher cell viability (*p* < 0.05) than MZCNOs and CZCNOs hydrogels. This could be due to the enhanced tensile strength, lower degradation, higher hydrophobicity, and crosslinking density of UZCNOs hydrogel synthesized by acoustic cavitation method. The cell viability results revealed that the percentage of cell viability was dependent on the fabrication method of hydrogels. Moreover, cell viability suggests that UZCNOs composite hydrogels would useful as potential drug transporters with good cytocompatibility. 

The LIVE/DEAD results suggest that the cell growth was considerably enhanced with f-CNOs inclusion and cells were extensively attached on the surface of the composite hydrogels. This could be due to the excellent cytocompatibility behavior and less degradability of f-CNOs. Overall, UZCNOs hydrogels exhibited better cell growth than a controller, MZCNOs, and CZCNOs samples. The LIVE/DEAD measurements also suggest a positive effect of f-CNOs on the osteoblast cells. Moreover, spherical cellular morphology was also observed on the surface of the hydrogels due to a contact angle with the cell medium, surface morphology, and surface chemical interactions of cells with hydrogels. In addition, it is also posited that the homogeneous colloidal dispersion of f-CNOs within the hydrogel matrix and wrapping with zein produced strengthened zein/f-CNOs hydrogels with amenable cytocompatibility. Nonetheless, improved mechanical strength, admiral cytocompatibility, the pH-responsive sustained drug release, and good pH-sensitivity of UZCNOs hydrogels can be useful as potential drug transporters for oral colon delivery systems and cartilage tissue engineering.

## Figures and Tables

**Table 1:** Reaction conditions and fabrication methods of hydrogels.

Entry	Method	Zein/f-CNOs	Cross-Linker (GA)	Time (min.)	Hydrogel ^d^
1.	Conventional	(1.0 g/2.0 mg)	(1%, *w*/*w*)	180 ^a^	CZCNOs
2.	Microwave	(1.0 g/2.0 mg)	(1%, *w*/*w*)	40 ^b^	MZCNOs
3.	Acoustic cavitation (ultra-sonic)	(1.0 g/2.0 mg)	(1%, *w*/*w*)	30 ^c^	UZCNOs

^a^ The reaction mixture was stirred at 65 °C for 180 min; ^b^ 500 W of power was applied for 40 min; ^c^ The reaction mixture was ultra-sonicated for 30 min; ^d^ The homogeneous reaction mixture was poured into a glass petri dish and left to stand overnight for gel formation.
